# Association between Abdominal Aortic Calcification and Coronary Heart Disease in Essential Hypertension: A Cross-Sectional Study from the 2013–2014 National Health and Nutrition Examination Survey

**DOI:** 10.3390/jcdd11050143

**Published:** 2024-05-02

**Authors:** Lan He, Xu Li, E Shen, Yong-Ming He

**Affiliations:** 1 Division of Cardiology, The First Affiliated Hospital of Soochow University, 188 Shizi Ave., Gusu District, Suzhou 215006, China; helan0325@163.com (L.H.); xu_li1996@163.com (X.L.); 2 Department of Ultrasound, Shanghai Chest Hospital, School of Medicine, Shanghai Jiao Tong University, 241 Huaihai West Road, Xuhui District, Shanghai 200030, China

**Keywords:** abdominal aortic calcification, coronary heart disease, essential hypertension

## Abstract

Background: This study aimed to investigate the association between abdominal aortic calcification (AAC) and coronary heart disease (CHD) in essential hypertension (EH). Methods: This study included patients diagnosed with EH during the 2013–2014 NHANES survey cycle. The study cohort was categorized into the following four groups based on their AAC-24 score: no AAC (0); mild AAC (1–4); moderate AAC (5–15); and severe AAC (16–24). Logistic regression models were used to assess the association between AAC and CHD. Restricted cubic spline curves (RCS) were used to explore possible nonlinear relationships between AAC and CHD. Results: The prevalence of CHD was found to be higher in the moderate AAC and severe AAC groups than in the group without AAC (40.1% versus 30.9%, 47.7% versus 30.9%). On a continuous scale, the fully adjusted model showed a 7% increase in the risk of CHD prevalence per score increase in AAC [OR (95% CI) = 1.07 (1.03–1.11)]. On a categorical scale, the fully adjusted model showed the risk of CHD prevalence in EH patients with moderate AAC and severe AAC was 2.06 (95%CI, 1.23–3.45) and 2.18 (1.09–5.25) times higher than that in patients without AAC, respectively. The RCS curve suggested a dose-response linear relationship between AAC and CHD. Conclusion: These findings highlight that in patients with EH, a higher severity of AAC is associated with a higher risk of CHD prevalence.

## 1. Introduction

Arterial calcification, a primary indicator of arterial stiffness, is strongly correlated with cardiovascular disease risk [1]. It has been unequivocally identified as a predictor for coronary heart disease (CHD) [2,3]. Commonly-used clinical measures of arterial calcification include coronary artery calcification (CAC) and abdominal aortic calcification (AAC) [4]. AAC is increasingly recognized due to its easier localization and quantification, as well as its earlier occurrence compared to CAC [5,6]. Serving as a sensitive predictor of CHD, AAC has been shown to outperform the Framingham risk score [7]. In a 5-year prospective study, patients with AAC scores above 5.5 exhibited a significantly higher incidence of CHD compared to those with AAC scores below 5.5 [8]. Jurgens et al. found that blacks tended to have higher AAC scores than whites, particularly among black women, which may help explain their disparate rates of CHD (3.6% for black women vs. 1.6% for white women) [9].

Hypertension represents a significant subgroup vulnerable to CHD [10,11]. Research indicates that approximately 71.8% of CHD patients also have comorbid hypertension, while about 30% of hypertensive patients attending outpatient clinics are concurrently diagnosed with CHD [12]. Moreover, the mortality rate from CHD is 2.3 times higher in the presence of hypertension [13]. Additionally, studies have revealed that hypertensive patients constitute the primary demographic affected by arterial calcification [14]. This susceptibility arises from the pathological changes characteristic of hypertension [15,16,17]. However, to our knowledge, the relationship between AAC and CHD in hypertensive patients has not been previously explored. Therefore, the objective of this study was to examine the association between AAC and CHD in essential hypertension (EH) using the cross-sectional design.

## 2. Methods

### 2.1. NHANES

NHANES, the National Health and Nutrition Examination Survey, is a cross-sectional, population-based survey designed to collect information about the health and nutrition status of the U.S. household population. The program surveys a nationally representative sample of approximately 5000 individuals each year, located in counties across the country, to represent the U.S. population. NHANES includes both an interview, which includes questions on demographics, socioeconomics, diet, and health, and a physical examination, which includes physiologic measurements, laboratory tests, and other components. The National Institutes of Health Research Ethics Review Board approved the NHANES survey protocol, and all participants signed and provided informed consent. NHANES data are publicly available from the official website [18].

### 2.2. Study Population

This study included patients diagnosed with EH during the 2013–2014 NHANES survey cycle. EH was defined as SBP ≥ 140 and/or DBP ≥ 90 mmHg or as a self-reported one by asking the question, “Has a doctor or other health professional ever told you that you have hypertension?” The exclusion criteria were as follows: (i) age < 18; (ii) without EH or missing data on EH; and (iii) missing data on AAC or CHD. The degree and extent of AAC were assessed using the AAC-24 semi-quantitative technique (Kauppila, 1997; Schousboe, 2007) [19,20]. The AAC score was extracted from the examination data for NHANES. Lateral spine images were acquired by dual-energy X-ray absorptiometry. Lateral spine images were analyzed in the region of L1–L4 vertebrae, which were divided into 4 segments with the midpoint of the intervertebral space as the boundary. The total calcification score of the anterior and posterior walls of the corresponding vessels was calculated for each spinal segment. The study population was divided into the following 4 groups according to the AAC-24 score: without AAC (0); mild AAC (1–4); moderate AAC (5–15); and severe AAC (16–24).

### 2.3. Study Outcome

The outcome of this study was CHD prevalence, which was self-reported by asking the question, “Have you been told by a doctor that you have CHD?”. CHD included all chronic coronary artery disease (stable angina, ischemic cardiomyopathy, and occult coronary artery disease) and acute coronary syndromes (unstable angina, non-ST-segment elevation myocardial infarction, and ST-segment elevation myocardial infarction). 

### 2.4. Covariates

Age, sex, ethnicity, diabetes mellitus (DM), smoking, and drinking were obtained through questionnaires. The diagnosis of diabetes mellitus (DM) referred to the most recent ADA criteria (FPG ≥ 7.0 mmol/L, A1C ≥ 6.5%, 2-h OGTT ≥ 11.1 mmol/L, or a random plasma glucose ≥ 11.1 mmol/L) [21]. The definitions of smoking and drinking refer to the latest standards on the New Zealand Ministry of Health website [22]. Body mass index (BMI) and pulse were obtained by physical examination, where BMI was evaluated by body mass (kilograms) and body height (m^2^). Red blood cells (RBCs), white blood cells (WBCs), creatinine, triglycerides, low-density lipoprotein cholesterol (LDL-C), and high-density lipoprotein cholesterol (HDL-C) were obtained by laboratory measurements. Blood cell counts and hemoglobin levels were analyzed with the Beckman–Coulter MAXM or DXH 800. Albumin and creatinine were measured using the DcX800 method. Triglycerides and HDL-C were analyzed by the Roche/Hitachi Modular P Chemistry Analyzer (Mod P) in Mobile Examination Centers (MECs). LDL-C was calculated from measured values of triglycerides, HDL-C, and TC according to the Friedewald algorithm. Details on the methods are publicly available on the official NHANES website [18].

### 2.5. Statistical Analysis

All analyses were performed using Stata 17 (Stata Corp., Texas, USA). Multiple imputations (chained equations, 25 times) were used to fill in the missing values for all covariates. A trend test was used for the comparison of baseline characteristics between independent groups. Logistic regression models were used to assess the association between AAC and CHD. The model was progressively adjusted as follows: Model 1, unadjusted; Model 2, adjusted for age, sex, and ethnicity; Model 3, further adjusted for BMI, pulse, drinking, smoking, and DM; and Model 4, further adjusted for RBC, WBC, platelets, albumin, creatinine, triglyceride, LDL-C, and HDL-C. Restricted cubic spline curves (RCS) were used to explore possible nonlinear relationships between AAC and CHD. Subgroup analyses were performed to test whether the association between AAC and CHD was consistent in different subgroups. Sensitivity analyses tested whether the model was affected by treatment with antihypertensive drugs. All tests were two-sided. Statistical significance was considered when a *p* < 0.05.

## 3. Results

### 3.1. Baseline Characteristics

**Figure 1:**
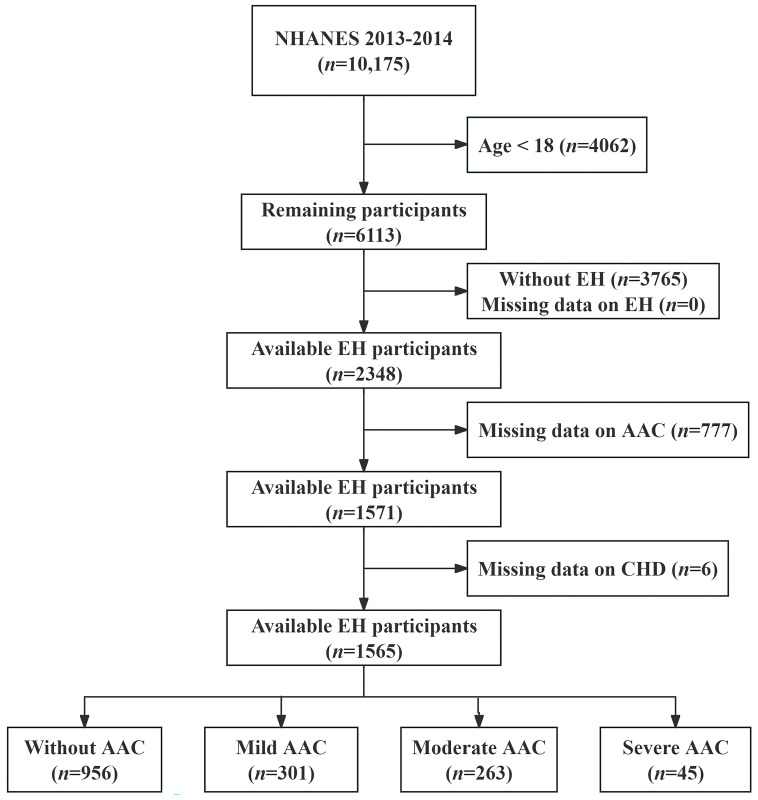
Flowchart of participant selection. Abbreviations: EH = essential hypertension; AAC = abdominal aortic calcification; and CHD = coronary heart disease.

A total of 10,175 participants were initially included for potential analyses during the 2013–2014 NHANES survey cycle. After excluding participants who did not have essential hypertension (EH), were underage, or had missing data on CHD or AAC, 1565 patients with EH were finally analyzed in this study. Among these EH patients, there were 956 without AAC, 301 with mild AAC, 263 with moderate AAC, and 45 with severe AAC, as depicted in Figure 1. The prevalence of CHD was higher in the moderate AAC and severe AAC groups compared to the group without AAC (40.1% versus 30.9%, 47.7% versus 30.9%), as indicated in Table 1. Furthermore, EH patients with severe AAC were older, more likely to be former smokers, had a lower BMI, and had a higher prevalence of type 2 diabetes mellitus.

### 3.2. Association between AAC and CHD in EH on a Continuous Scale

On a continuous scale, each one-point increase in the AAC-24 score was associated with a 13% increase in the risk of coronary heart disease (CHD) prevalence, with an odds ratio (OR) of 1.13 (95% confidence interval [CI], 1.09–1.17) in the unadjusted model. This association remained significant even after adjusting for a broad spectrum of variables in the multivariable analysis. More detailed information can be found in Table 2.

### 3.3. Association between AAC and CHD in EH on a Categorical Scale

**Figure 2:**
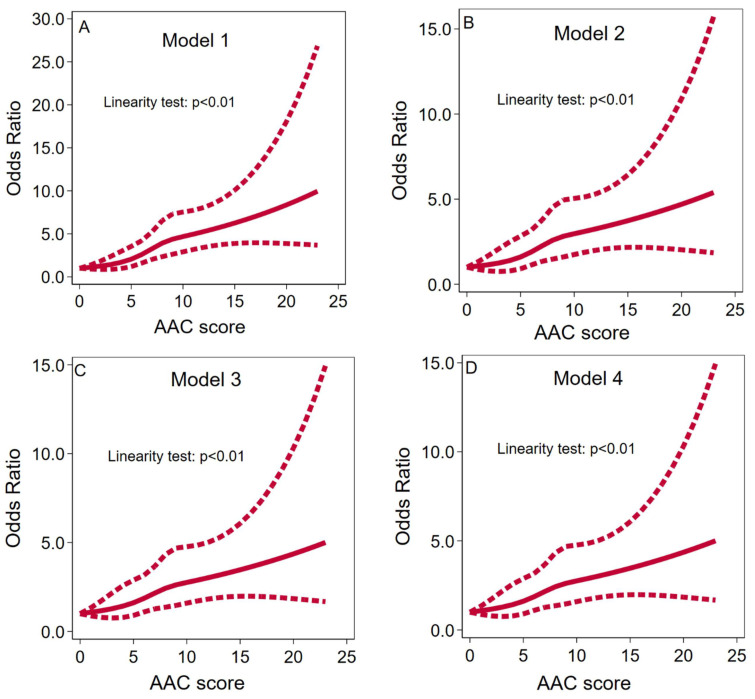
RCS curves for examining the nonlinear relationship between AAC score and CHD.

On a categorical scale, the results of the univariate analysis showed that the risk of coronary heart disease (CHD) prevalence was 1.26 (95% confidence interval [CI], 0.74–2.15) times higher for mild AAC, 3.96 (2.60–6.04) times higher for moderate AAC, and 5.74 (2.75–11.99) times higher for severe AAC, compared to individuals without AAC. The *p*-value for the trend was less than 0.001, indicating a significant relationship between AAC severity and CHD risk. These associations and trends remained significant even after adjusting for a broad spectrum of variables in the multivariable analysis. More information can be found in Table 2. The restricted cubic spline (RCS) curve analysis indicated a linear dose–response relationship between AAC severity and CHD risk in individuals with essential hypertension (EH), rather than a nonlinear relationship, as depicted in Figure 2.

### 3.4. Subgroup and Sensitivity Analysis

**Figure 3:**
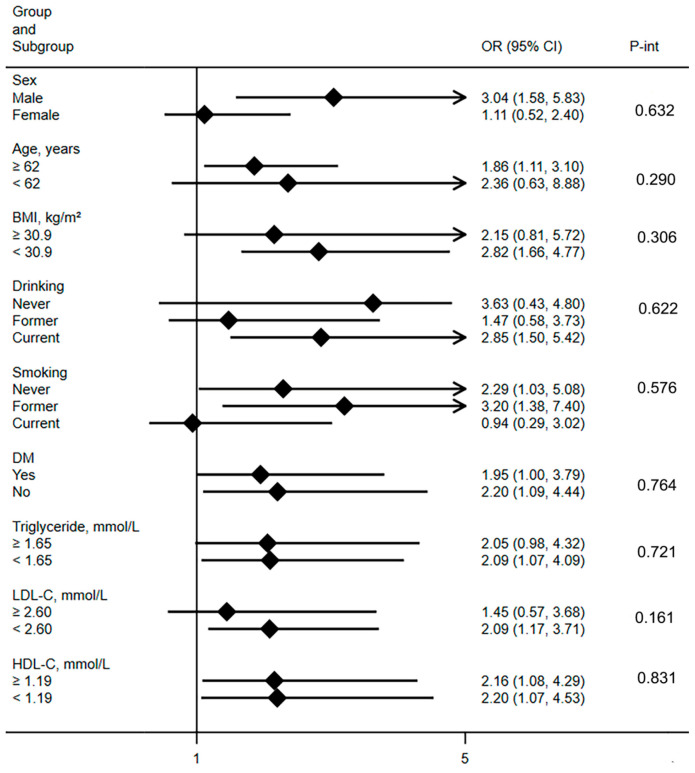
The results showed the OR (95% CI) of moderate AAC in different subgroups when without AAC was used as a reference. Subgroup analysis was adjusted for all variables as shown in Model 4 in Table 2. Abbreviations: OR = odds ratio; CI = confidence interval; and P-int = P-interaction.

Subgroup analysis largely corroborated the associations between AAC and CHD in EH uncovered in the current study across a broad spectrum of risk factors, as depicted in Figure 3. The use of antihypertensive drugs may potentially influence abdominal aortic calcification. Therefore, we conducted a sensitivity analysis specifically within the population taking antihypertensive drugs. This did not alter our main findings, as demonstrated in Table 3. Additionally, our stratified analysis based on hypertension severity did not alter the results. Details can be found in Supplementary Tables S1 and S2.

## 4. Discussion

To the best of our knowledge, this is the first study examining the association between AAC and CHD in EH. The key findings of this study are that (i) a higher severity of AAC is associated with a higher risk of CHD prevalence in EH patients; and (ii) this association is displayed in a dose–response manner.

Arterial calcification stands out as a robust and independent risk factor for CHD [23]. This calcification process involves the deposition of hydroxyapatite crystals within the vasculature. Within the arterial wall, vascular smooth muscle cells (VSMCs) constitute the primary cell type, while elastin emerges as the predominant protein [24]. When the vascular wall is under oxidative stress [25], inflammation [26], apoptosis, aging [27], etc., the VSMC transdifferentiates to osteoblasts, and the elastin is lost in large quantities, which increases the expression of osteogenesis-related factors RUNX2 and BMP2 [28] and increases the occurrence of vascular calcification. Consequently, vascular calcification ensues, escalating arterial stiffness, pulse pressure, and left ventricular hypertrophy, culminating in CHD [29]. Our study underscores a significant association between AAC and CHD in patients with EH. In asymptomatic chronic dialysis patients, multifactorial regression analysis unveiled the AAC score as an independent predictor of CAD presence, with a 1.18-fold increased CAD risk per 1-point AAC score elevation [OR (95%CI), 1.18 (1.06–1.32)] [30]. Similarly, in type 2 diabetics, the prevalence of CHD was found to be twice as high in patients with AAC > 0 compared to those with AAC = 0 (28% versus 14%). Our study echoed these findings, revealing that patients with AAC (mild, moderate, and severe AACs) exhibited a 2.23-fold higher prevalence of CHD compared to those without AAC (69.1% versus 30.9%). Extensive research has underscored AAC as a significant cardiovascular disease risk factor in diabetic patients [31]. Parikh and colleagues observed a 2.1-fold increase in the odds of AAC in individuals with early-onset CHD within the third-generation population of the Framingham Heart Study, further supporting the association between AAC and heightened CHD risk [32]. Our study, encompassing a non-selective study population, bolsters the credibility of AAC-CHD associations. Additionally, while prior studies did not unveil the relationship between AAC and CHD in EH, a sole study found AAC to be independently linked to total CHD mortality in community-dwelling individuals [33]. Our findings suggest a linear association between AAC and CHD in EH, with elevated AAC scores correlating with heightened CHD prevalence.

### Limitations

There are several limitations that warrant attention when interpreting our findings, as follows: (i) This study adopts the cross-sectional design, underscoring the necessity for further randomized, controlled trials to validate the relationship between AAC severity and CHD incidence. (ii) Certain factors potentially linked to coronary heart disease, such as sleep duration, exercise habits, thrombophilia, and cardiac arrhythmia, were not accounted for in our analysis. (iii) Some of the variables considered in this study, including smoking, alcohol consumption, hypertension, and diabetes mellitus, relied on subjective questionnaires, introducing the potential information bias.

## 5. Conclusions

In patients with EH, a higher severity of AAC is associated with a higher risk of CHD prevalence.

## Figures and Tables

**Table 1:** Baseline characteristics stratified by AAC severity.

Factor	Without AAC (*n* = 956)	Mild AAC (*n* = 301)	Moderate AAC (*n* = 263)	Severe AAC (*n* = 45)	*p* for Trend
Age, year	59.87 ± 10.86	64.79 ± 10.07	70.66 ± 9.57	74.40 ± 6.69	<0.001
Female	486 (52.4%)	152 (51.9%)	136 (52.9%)	24 (54.5%)	0.842
Ethnicity					0.665
Mexican American	118 (12.7%)	30 (10.2%)	20 (7.8%)	0 (0.0%)	
Non-Hispanic White	340 (36.7%)	147 (50.2%)	149 (58.0%)	30 (68.2%)	
Non-Hispanic Black	274 (29.6%)	59 (20.1%)	41 (16.0%)	7 (15.9%)	
Non-Hispanic Asian	93 (10.0%)	30 (10.2%)	28 (10.9%)	4 (9.1%)	
Others	102 (11.0%)	27 (9.2%)	19 (7.4%)	3 (6.8%)	
BMI, kg/m^2^,	30.12 ± 6.05	28.43 ± 5.15	27.55 ± 4.67	27.16 ± 4.08	<0.001
Pulse, beats/min	72.11 ± 11.83	71.61 ± 12.24	69.23 ± 10.77	69.80 ± 11.72	<0.001
Drinking					0.806
Never	151 (16.3%)	44 (15.0%)	35 (13.6%)	6 (13.6%)	
Former	200 (21.6%)	67 (22.9%)	72 (28.0%)	16 (36.4%)	
Current	576 (62.1%)	182 (62.1%)	150 (58.4%)	22 (50.0%)	
Smoking					0.002
Never	521 (56.2%)	143 (48.8%)	111 (43.2%)	18 (40.9%)	
Former	251 (27.1%)	98 (33.4%)	87 (33.9%)	20 (45.5%)	
Current	155 (16.7%)	52 (17.7%)	59 (23.0%)	6 (13.6%)	
DM	286 (30.9%)	91 (31.1%)	103 (40.1%)	21 (47.7%)	0.001
RBC, 10^3^/μL	4.62 ± 0.48	4.58 ± 0.487	4.44 ± 0.49	4.23 ± 0.58	<0.001
WBC, 10^3^/μL	7.18 ± 2.14	7.37 ± 2.11	7.37 ± 2.08	7.56 ± 2.28	0.217
Platelets, 10^3^/μL	233.82 ± 59.27	225.16 ± 59.05	220.05 ± 56.12	213.40 ± 62.13	<0.001
Albumin, g/L	42.12 ± 3.18	42.19 ± 3.19	41.70 ± 3.18	42.56 ± 3.53	0.381
Creatinine, umol/L	85.62 ± 44.34	90.48 ± 88.84	97.95 ± 56.14	106.52 ± 83.69	<0.001
Triglycerides, mmol/L	1.50± 0.89	1.51 ± 0.77	1.51 ± 0.73	1.44 ± 0.75	0.484
LDL-C, mmol/L	2.94 ± 0.96	2.92 ± 1.02	2.75 ± 0.95	2.53 ± 0.92	<0.001
HDL-C, mmol/L	1.39 ± 0.43	1.35 ± 0.41	1.36 ± 0.38	1.39 ± 0.54	0.373
CHD	286 (30.9%)	91 (31.1%)	103 (40.1%)	21 (47.7%)	<0.001

Notes: continuous and categorical variables were presented as mean ± SD or percentages n (%), respectively. BMI = body mass index; DM = diabetes mellitus; RBC = red blood cell; WBC = white blood cell; LDL-C = low-density lipoprotein cholesterol; HDL-C = high-density lipoprotein cholesterol; and CHD = coronary heart disease.

**Table 2:** ORs and 95% confidence intervals for the association between AAC and CHD in EH.

AAC Score	Model 1	Model 2	Model 3	Model 4
	OR (95% CI)	OR (95% CI)	OR (95% CI)	OR (95% CI)
Continuous per score increase	1.13 (1.09–1.17)	1.09 (1.05–1.13)	1.08 (1.04–1.12)	1.07 (1.03–1.11)
Categorical per unit increase				
Without AAC (0)	1 (Ref.)	1 (Ref.)	1 (Ref.)	1 (Ref.)
Mild AAC (1–4)	1.26 (0.74–2.15)	1.03 (0.59–1.78)	1.08 (0.61–1.88)	1.09 (0.61–1.95)
Moderate AAC (5–15)	3.96 (2.60–6.04)	2.47 (1.53–3.97)	2.24 (1.37–3.67)	2.06 (1.23–3.45)
Severe AAC (6–24)	5.74 (2.75–11.99)	2.93 (1.29–6.59)	2.62 (1.13–6.05)	2.18 (1.09–5.25)

Model 1: unadjusted. Model 2: adjusted for age, sex, and ethnicity. Model 3: further adjusted for BMI, pulse, drinking, smoking, and DM. Model 4: further adjusted for RBC, WBC, platelets, albumin, creatinine, triglyceride, LDL-C, and HDL-C. Abbreviations: AAC = abdominal aortic calcification; OR = odds ratio; and CI = confidence interval.

**Table 3:** ORs and 95% confidence intervals for the association between AAC and CHD in EH patients taking antihypertensive drugs.

AAC Score	Model 1	Model 2	Model 3	Model 4
	OR (95% CI)	OR (95% CI)	OR (95% CI)	OR (95% CI)
Continuous per score increase	1.10 (1.08–1.15)	1.08 (1.04–1.12)	1.07 (1.0–1.12)	1.06 (1.02–1.11)
Categorical per unit increase				
Without AAC (0)	1 (Ref.)	1 (Ref.)	1 (Ref.)	1 (Ref.)
Mild AAC (1–4)	1.16 (0.64–2.08)	1.04 (0.57–1.91)	1.05 (0.57–1.94)	1.08 (0.57–2.02)
Moderate AAC (5–15)	3.78 (2.40–5.96)	2.64 (1.57–4.41)	2.36 (1.38–4.04)	2.32 (1.33–4.06)
Severe AAC (6–24)	5.58 (2.60–11.97)	3.25 (1.39–7.58)	2.83 (1.19–6.74)	2.50 (1.01–6.19)

Model 1: unadjusted. Model 2: adjusted for age, sex, and ethnicity. Model 3: further adjusted for BMI, pulse, drinking, smoking, and DM. Model 4: further adjusted for RBC, WBC, platelets, albumin, creatinine, triglyceride, LDL-C, and HDL-C. Abbreviations: AAC = abdominal aortic calcification; OR = odds ratio; and CI = confidence interval.

## Data Availability

All data were obtained from the NHANES database (https://wwwn.cdc.gov/nchs/nhanes/Default.aspx, accessed on 15 June 2023).

## Supplementary Materials

The following supporting information can be downloaded at: https://www.mdpi.com/article/10.3390/jcdd11050143/s1. Table S1: Definition and grading of hypertension; Table S2: The association between AAC and CHD after stratification by the grade of hypertension.
