# Supplementary material for: Association between Abdominal Aortic Calcification and Coronary Heart Disease in Essential Hypertension: A Cross-Sectional Study from the 2013–2014 National Health and Nutrition Examination Survey
Source: J Cardiovasc Dev Dis. 2024 May 2;11(5):143. doi: 10.3390/jcdd11050143 (PMC11122146; doi:10.3390/jcdd11050143)
Supplement: Supplementary file 1 [file jcdd-11-00143-s001.zip › jcdd-2955721-supplementary.pdf]

Table S1: Definition and grading of hypertension

Defining characteristic	The grade of hypertension	Cases
The average of three blood pressure measurements	Grade 1: 40≤SBP<160 and/or 90≤ DBP<100	488
	Grade 2: 60≤SBP<180 and/or 100≤ DBP<110	130
	Grade 3: 0≤SBP and/or 110≤ DBP	41
Self-reported hypertension	Not applicable	906

Table S2: The association between AAC and CHD after stratification by the grade of hypertension.

AAC score	Grade 1	Grade 2	Grade 3
	OR (95% CI)	OR (95% CI)	OR (95% CI)
Continuous per score increase	1.08(1.09-1.17)	1.12(1.02-1.23)	1.15(1.04-1.38)
Categorical per unit increase			
Without AAC (0)	1(Ref.)	1(Ref.)	1(Ref.)
Mild AAC (1-4)	1.84(0.77-4.39)	1.90(0.39-9.09)	2.44(0.61-1.88)
Moderate AAC (5-15)	2.25(1.87-5.81)	4.12(1.11-16.84)	5.55(1.48-25.67)
Severe AAC (6-24)	4.05(2.81-20.3)	8.25(1.24-59.04)	16.82(1.57-85.01)

Model adjusted for age, sex, ethnicity, BMI, pulse, drinking, smoking, DM, RBC, WBC, platelets, albumin, creatinine, triglyceride, LDL-C, and HDL-C.
